# The Role of Serum Vitamin D Levels and Vitamin D Receptor (*VDR*) Gene Variants on Dental Caries

**DOI:** 10.3390/children12010007

**Published:** 2024-12-24

**Authors:** Ece Şengün Berber, Feyza Umay Koç, Ayça Aykut, Burcu Barutçuoğlu, Fahinur Ertuğrul, Merve Tosyalı, Mert Pekerbaş, Arzu Aykut Yetkiner

**Affiliations:** 1 Department of Dentistry Services, Vocational School of Health Services, Dokuz Eylul University, Izmir 35330, Turkey; ece.sengunberber@deu.edu.tr; 2 Department of Pedodontics, Faculty of Dentistry, Ege University, Izmir 35040, Turkey; fahinur.ertugrul@ege.edu.tr (F.E.); arzu.yetkiner@ege.edu.tr (A.A.Y.); 3 Department of Social Pediatrics, Faculty of Medicine, Ege University, Izmir 35040, Turkey; merve.tosyali@ege.edu.tr; 4 Department of Medical Genetics, Faculty of Medicine, Ege University, Izmir 35040, Turkey; ayca.aykut@ege.edu.tr (A.A.); mertpekerbas@gmail.com (M.P.); 5 Department of Clinical Biochemistry, Faculty of Medicine, Ege University, Izmir 35040, Turkey; burcu.barutcuoglu@ege.edu.tr

**Keywords:** dental caries, vitamin D deficiency, 25-hydroxyvitamin D, *VDR* gene variants

## Abstract

Background/Objectives: Vitamin D helps the mineralization of bone, teeth, and other calcified tissues by regulating calcium–phosphate metabolism. The nuclear activation of the vitamin D receptor (*VDR*) gene is essential for the effectiveness of vitamin D. The main objective of this study is to determine the role of vitamin D levels and *VDR* gene variants in dental caries. Methods: This study included 128 3–6-year-old children who were divided into 64 caries-free and 64 carious children. Blood samples were taken, and serum 25-hydroxyvitamin D levels were measured. Intraoral examinations were performed to record dmft index values. Genetic testing was performed on 26 children to examine VDR gene variations. Relevant gene regions were amplified using PCR and sequenced via Sanger sequencing in a total of 13 caries-free and 13 carious children. Stats analyses included chi-square/trend tests to compare count data; Student’s T/Mann–Whitney U tests for two-group normally/non-normally distributed data; and the Kruskal–Wallis test for 3+ groups with non-normal data. Results: The results showed that vitamin D levels were relatively low in the caries group, but no statistically significant relationship was found between vitamin D levels and caries. No mutations were detected in the VDR gene in either group, and there was no significant difference observed in terms of the number of ApaI, TaqI, and FokI polymorphisms. Based on these findings, the use of prophylactic vitamin D supplements to prevent caries formation or development cannot be recommended. Conclusions: This study provides important insights into the relationship between vitamin D and dental caries and can contribute to the development of effective preventive strategies for oral and dental health.

## 1. Introduction

Oral health is a public health issue that is closely related to the general health and quality of life of people, with dental caries being one of the main problems affecting oral and dental health [1,2]. Dental caries is a common, chronic, and infectious disease that involves microbial, behavioral, genetic, and environmental factors [2,3].

Vitamin D, a prohormone, has endocrine, autocrine, and paracrine activities. It also plays an essential role in calcium (Ca^+2^) homeostasis and bone metabolism, as well as a positive role in the absorption and utilization of calcium–phosphorus (Ca^+2^-P^−3^), which is effective in accumulating in mineralized tissues [4,5]. Vitamin D has been reported to be associated with immunity, cognitive function, blood pressure regulation, cardiometabolic conditions, and aging [4,6].

Vitamin D is an essential element for the regulation of serum Ca^+2^, P^−3^, and alkaline phosphatase (ALP). By maintaining the balance between vitamin D and these minerals, both bone turnover and Ca^+2^-P^−3^ hemostasis are preserved [7,8]. Measurements of serum ALP, Ca^+2^, and P^−3^ values are frequently examined in routine biochemical measurements [9]. In addition, the concentration of hemoglobin (HMG), which binds and transports oxygen in the blood, is another frequently examined biochemical parameter [10].

Circulating serum 25-hydroxyvitamin D (calcidiol, 25(OH)D) levels are the best indicator of total vitamin D from both endogenous and exogenous sources [11]. The main method used to determine vitamin D deficiency is the measurement of serum 25(OH)D levels since its half-life is approximately three weeks, and its plasma concentration can be measured in a relatively high amount compared to 1,25-dihydroxy vitamin D (calcitriol, 1,25(OH)_2_D), which is the active form of vitamin D [12].

Vitamin D performs its intracellular biological functions by binding to its intracellular receptor. Due to genetic changes in the structure of the vitamin D receptor (*VDR*) gene and changes in protein fibers, there are positive effects on Ca^+2^ metabolism, cell proliferation, and the consumption system [13].

Although various studies have shown that tooth mineralization problems are more common in individuals with vitamin D deficiency, the results are inconsistent. In these studies, it has been shown that due to high serum 25(OH)D levels with vitamin D supplementation, caries risk can be reduced [4,14,15,16,17].

Although investigating the effects of vitamin D on general health is still a subject of research, the current literature review did not find a sufficient number of studies demonstrating the direct effect of vitamin D levels on dental caries.

The aim of this study is to investigate the relationship between dental caries, which is still an important public health problem, and 25(OH)D levels, and *VDR* gene variants and to show whether vitamin D can be used as a potential agent against dental caries.

## 2. Materials and Methods

### 2.1. Study Design and Participants

This study was conducted in accordance with the guidelines of the World Medical Association Declaration of Helsinki, and informed consent was obtained from all children’s parents.

This study was conducted at the Ege University Faculty of Medicine Children’s Hospital from December 2021 to March 2022. 

### 2.2. Sample Size Determination

The sample size for the biochemical part of this study was determined based on a power analysis performed by the Ege University Faculty of Medicine Department of Biostatistics and Medical Informatics. An independent samples *t*-test (Student’s *t*-test) was used to compare serum 25(OH)D levels between the caries-free and caries groups. It was determined that approximately 64 children per group would be required to achieve an alpha of 0.05 and a power of 80% (type 1 error rate) with a medium effect size (d = 0.5) between the two groups.

In this study, we included 128 children between the ages of 3 and 6 who were systemically healthy, had no physical disabilities, and had not undergone routine blood tests in the last year. Of these children, 64 had caries, and 64 were caries-free. The participants and their families were recruited from the Ege University Faculty of Medicine Department of Child Health and Diseases and Ege University Faculty of Medicine Children’s Hospital Dentistry Clinic for treatment and control. Patients were selected randomly, and the gender distribution was balanced.

This study excluded children with chronic diseases affecting vitamin D levels (e.g., obesity, type 1 diabetes, celiac disease, asthma, hypertension), endocrine, cardiovascular, or nephrological disorders (e.g., Turner syndrome, hypothyroidism, renal insufficiency), children under 18 months or over 72 months of age, those with developmental dental malformations, and children with mental disabilities.

For genetic analyses, blood samples were taken from the participants who consented to genetic analysis, and a complete gene analysis was performed on a total of 26 children, with 13 children from each group (caries and caries-free).

### 2.3. Data Collection

#### 2.3.1. Dental Examination and Questionnaire

All individuals were subjected to an oral and dental examination at the Ege University Faculty of Medicine, Children’s Hospital, Dentistry Clinic using an intraoral mirror and examination probe in the dental unit under reflector light by the same researcher to ensure standardization of the inspections. dmft scores were assigned according to the guidelines of the World Health Organization (1997).

A questionnaire was prepared to assess each child’s personal history, eating habits, knowledge of vitamin D, oral health, and socio-economic status. Families were asked about their children’s daily sugar consumption, whether they had professional fluoride treatments, and their daily use of fluoride toothpaste. The consumption of animal foods containing vitamin D, such as milk, cheese, eggs, and fish, was also queried. Responses were grouped into those who consume at least one of these foods every day, those who do not consume at least two of these foods, and those who rarely consume these products. The use of daily prophylactic vitamin D or multivitamins containing vitamin D was also investigated. The questionnaire was filled out by the families of the children in both groups.

#### 2.3.2. Biochemical Tests

In the first clinical part of this study, venous blood sampling was performed on all patients (*n* = 128) by taking three tubes of blood. Routine laboratory methods were used to determine the serum Ca^+2^, ALP, P^−3^, and 25(OH)D levels and complete blood count (CBC).

- A total of 5 cc of blood to analyze Ca^+2^, ALP, and P^−3^ levels was collected into yellow top tubes containing serum separating gel.
- A total of 2 cc of blood for CBC analysis was collected into purple top tubes containing K2-EDTA. Hemoglobin (HGB) values were recorded from the CBC tests.
- A total of 2 cc of blood for vitamin D measurement was collected into purple top tubes containing K2-EDTA.

The liquid chromatography–tandem mass spectrometry (LC/MS) analysis method using Agilent 6460 Triple Quadripole (Agilent Technologies Inc., Santa Clara, CA, USA) was used to evaluate the serum 25(OH)D levels of all children. 

A literature review revealed various classifications for vitamin D reference values. Our study used commonly cited reference ranges (including both optimal and suboptimal values) for the main evaluation. We also included reference levels determined by the laboratory, which combine these ranges. This approach aimed to provide a comprehensive assessment and identify any differences. Since no significant differences were found, the values were consolidated. Serum vitamin D levels were recorded according to two different reference ranges, which are shown in Table 1.

#### 2.3.3. Genetic Analysis

In the second clinical part of this study, for the genetic analyses, blood samples were taken from the participants who consented to genetic analysis, and a complete gene analysis was performed. Genetic analysis was performed on a total of 26 children; with 13 children from each group, the group numbers came out randomly equal.

To investigate pathogenic variations (mutations and polymorphisms) in the *VDR* gene, one tube of venous blood was sampled from all patients. 

According to the Guidelines on Ethical Approaches in Clinical Trials in the Pediatric Population, the amount of blood taken in children should not exceed 0.8 milliliters/kilogram (mL/kg) at one time [21]. Therefore, 2 cc of blood for genetic analysis was collected into purple top tubes containing K2-EDTA and sent to the Molecular Genetics Laboratory at the Medical Genetics Department for the analysis of *VDR* gene variants using PCR and agarose gel imaging.

The *VDR* gene variations analysis involved sequencing of the coding exons and the exon–intron junctions of the entire gene. Genomic DNA was extracted from blood cells using standard techniques with the QIAcube Connect and QIAcube AllPrep DNA/RNA FFPE kit (Product No:80234, QIAGEN, Hilden, Germany). The quantity and quality of the obtained DNA samples were analyzed spectrophotometrically using ThermoScience NanoDrop™ One/OneC (Thermo Fisher Scientific, Waltham, MA, USA). The exons of the *VDR* gene were amplified using the primers listed in Table 2, and all PCR products were sequenced by the dye termination method using a DNA sequencing kit (Perkin-Elmer, Foster, CA, USA) and analyzed using the ABI Prism 3100 sequence analyzer (Applied Biosystems, Foster, CA, USA). The variations found were evaluated using the data in the Ensembl database and The Human Gene Mutation Database (HGMD).

### 2.4. Statistical Analysis

The biochemical and genetic data obtained in this study were analyzed using SPSS 25.0 (Statistical Product and Service Solutions, IBM, New York, NY, USA). The normality of the data was evaluated using the Kolmogorov–Smirnov test. For the comparison of count data between groups, chi-square and trend–presence chi-square trend tests were used. The Mann–Whitney test and *t*-test were used to compare two independent groups, while the Kruskal–Wallis test was used to compare more than two groups. A post hoc test was not performed since there was no significant difference between the groups. The level of significance was set at 0.05.

We used the chi-square test of independence to investigate the association between the identified genetic polymorphisms and dental caries.

## 3. Results

This study included a total of 128 children aged 3–6 years who visited the Ege University, Faculty of Medicine, Department of Child Health and Diseases, and Ege University, Faculty of Medicine, Children’s Hospital Dentistry Clinic, between 17 December 2021, and 3 March 2022, for either treatment or control. The gender distribution was equal, with 50% male and 50% female participants, and the median age of the group was 5 years (ranging from 3 to 6 years). Table 3 presents the characteristics of the children. The mean age of the 128 (64 males and 64 females categorized by caries status) children was 4.96 ± 1.068 (mean ± standard deviation) years.

In these children, dmft indexes were evaluated, and the mean dmft was 2.79 ± 3.46 (n = 128), and the median value was 0.50 (min. 0.00–max. 12.0). In this study, we found that caries levels increase with age. There was a significant correlation between age and caries status (*p* = 0.000). 

The data obtained as a result of the biochemical analysis of the children participating in this study are presented in Table 4. Table 4 presents the bivariate analysis results of the association between 25(OH)D levels and dental caries experience. The group of children with 25(OH)D levels lower than 30 ng/mL had a higher proportion of dental caries experience than the group of children with 25(OH)D levels of 30 ng/mL or more. In accordance with the three-category classification, children with severe vitamin D deficiency (0–9 ng/mL) showed a slightly higher proportion of dental caries experience compared to those with optimal levels (20–50 ng/mL), but this difference was not statistically significant. The mean 25(OH)D level was 20,41 (min. 3.00–maks. 50.00) in the caries-free group and 17.97 (min. 5.00–maks. 43.00) in the caries group. However, no statistically significant association was observed between vitamin D status and caries (*p* = 0.642).

The children who participated in this study were divided into separate groups based on their ages, and the relationship between the presence of caries and age was evaluated using Fisher’s exact test. There was no statistically significant difference found between the age groups (age 3 [*p* = 0.342], age 4 [*p* = 0.669], age 5 [*p* = 0.644], age 6 [*p* = 0.920]).

Table 5 presents the correlation between vitamin D and the other evaluated biochemical variables. When a correlation analysis was conducted to examine the relationship between vitamin D and the four biochemical measurements, including HGM, P^−3^, ALP, and Ca^+2^, there was no significant correlation found between them. When a correlation analysis was conducted to examine the relationship between age, dmft results, and vitamin D levels, no significant correlation was found.

In this study, sequencing was performed using the Sanger sequencing method for 13 caries-free and 13 caries-affected children, and the exon regions (3rd, 4th, 5th, 6th, 7th, 8th, 9th, and 10th) and exon–intron junctions were examined. No variant was detected in the examined regions except for known polymorphisms (Table 6). 

The third exon was analyzed in 25 children, and the FokI polymorphism was detected in 11 caries-free and 12 carious children. The 10th exon was analyzed in 26 children, and the TaqI polymorphism was detected in 10 caries-free and 10 carious children. The 10th exon and its exon–intron junctions were analyzed in 26 children, and the ApaI polymorphism was detected in 12 caries-free and 11 carious children.

**Figure 1:**
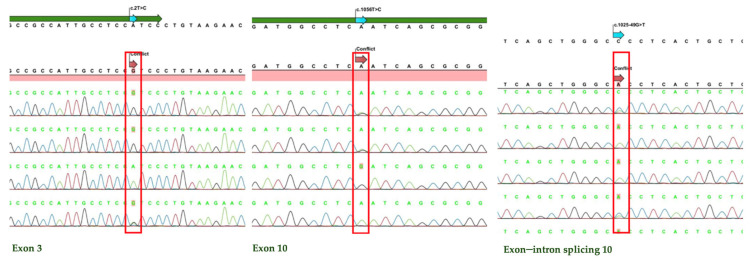
Illustration of examples of polymorphisms in exons and exon–intron junctions.

Figure 1 shows examples of the sequence analysis results. The green, black, and blue peaks represent the adenine, guanine, and cytosine bases, respectively. Peaks that match the reference genome indicate no polymorphism, while the presence of two differently colored peaks indicates a heterozygous polymorphism, and differently colored peaks from what is expected indicate a homozygous polymorphism in both alleles.

## 4. Discussion

In our study, we evaluated factors affecting dental caries formation through surveys and biochemical measurements, investigating the relationship between vitamin D levels and *VDR* gene variants. Our findings were compared with the existing literature in this context.

The half-life of the active form of vitamin D, 1,25(OH)_2_D, is known to be 3–6 h, while the half-life of the inactive form, 25(OH)D, is 3 weeks. The plasma concentration of 1,25(OH)_2_D is determined in pg/mL, while that of 25(OH)D is measured in ng/mL [22]. Therefore, in our study, serum 25(OH)D levels were measured using the tandem mass spectrometry method.

Although there is no universal standard for sufficient serum vitamin D levels, various studies have used different levels as a reference [15,19,23,24,25,26]. When examining studies in the literature, in most studies, a serum vitamin D level of 30 ng/mL is considered optimal, 20–30 ng/mL is considered insufficient (suboptimal), 10–20 ng/mL is considered deficient, and values below 10 ng/mL are considered severe [14,15,27]. These reference values were used as the primary reference values in this study, consistent with the literature.

'In our study, we conducted examinations based on the reference values used in similar studies and also the values used as a basis by the Faculty of Medicine at Ege University [16,28]. However, no statistically significant difference was found between the two reference ranges.

In our study, severe vitamin D deficiency was observed in 12.5% of all the children, while vitamin D insufficiency or deficiency was observed in 82% of the children. The mean vitamin D level was found to be 19.19 ng/mL. The other biochemical values measured were within the normal reference range for the majority of the children when compared to vitamin D levels. The prevalence of vitamin D deficiency has also been observed in various studies conducted in our country [9,29].

Vitamin D plays a critical role in normal bone mineralization and formation, starting from the development stages of the jaw and teeth. It is also believed to have continuing effects on dental caries even after tooth formation [30].

The effect of vitamin D on the Ca^+2^-P^−3^ mechanism is well-established, and vitamin D deficiency is a common cause of rickets in children, which impacts bone development and can lead to serious dental complications [31,32,33]. A study found that children with early childhood caries had low serum Ca^+2^ levels [34]. In our study, no directly measurable effect of vitamin D levels on serum Ca^+2^ and P^−3^ values was detected.

In our study, 128 systemically healthy children aged between 3 and 6 years were evaluated. As a result, it was observed that the dmft score increased with age. Considering factors such as the retention time of teeth in the mouth, this result was consistent with the literature and expected [35,36,37]. This situation can be attributed to the increased likelihood of encountering cariogenic factors as the time elapsed after tooth eruption increases.

Some studies have found a significant relationship between insufficient vitamin D levels (below 20 ng/mL) and dental caries [16,23]. In studies where 30 ng/mL was considered the optimal vitamin D level, vitamin D was suggested as a preventive factor for dental caries [11,25,34]. However, a study conducted in the United States reported a relationship between vitamin D and dental caries, though it noted the results were not convincing [26]. Our study found that 21.9% of the caries-free children had sufficient vitamin D levels, compared to 18% in the caries group, but this difference was not statistically significant. When considering 30 ng/mL as optimal, 21.9% of the caries-free group had optimal levels, while the caries group showed only 14.1%, though, again, no significant difference was found.

A study found a relationship between permanent tooth caries and vitamin D, but no significant association was found during the mixed dentition period [24]. Similarly, a study in the Netherlands showed a weak relationship between vitamin D deficiency and dental caries during the primary dentition period [38]. Special needs children with suboptimal vitamin D levels (below 20 ng/mL) had twice the caries rate, showing a significant link between early childhood caries and vitamin D deficiency in these children [39]. However, other studies found no significant relationship between vitamin D deficiency and dental caries [28,40,41,42].

A meta-analysis found that while some studies suggest a relationship between vitamin D and dental caries, establishing a direct significant link remains difficult [43]. Another review reported a weak association between vitamin D deficiency and dental pathologies, highlighting the need for more long-term studies [44]. Similar to these studies, our research found a trend suggesting a negative correlation between vitamin D levels and caries, but the relationship was not statistically significant, reinforcing the complexity of establishing a clear link.

In the second part of our study, *VDR* gene variations were examined. The active form of vitamin D, 1,25(OH)_2_D_3_, demonstrates its biological effect by binding to the *VDR* gene, which has a nuclear receptor [45]. Most tissues and cells in the body have the *VDR* gene. Many of them work with an enzymatic mechanism to convert 25(OH)D to 1,25(OH)_2_D [12,46].

The *VDR* gene regulates the mechanism of action of the vitamin D prohormone that controls bone and Ca^+2^ metabolism by controlling the transcription of different genes [47]. The *VDR* gene regulates the expression of genes that encode proteins responsible for maintaining Ca^+2^ balance upon binding with 1,25(OH)_2_D [48]. Patients with mutations in the *VDR* gene exhibit low serum Ca^+2^ and P^−3^ levels and high PTH and ALP levels. It has been reported that 1,25(OH)_2_D levels can be very high in these patients [49].

It has been reported that the *VDR* gene and vitamin D play a significant role in reducing the risk of many chronic diseases, including cancer, autoimmune diseases, diabetes, infectious diseases, metabolic syndrome, and cardiovascular diseases [12,46].

It has been reported that the *VDR* gene is highly polymorphic, with 470 SNP polymorphisms identified [46]. Among these, the commonly studied polymorphisms are FokI (rs2228570), BsmI (rs11544410), ApaI (rs7975232), and TaqI (rs731236) [50]. In a study conducted in the Turkish population, *VDR* gene polymorphisms were examined, highlighting the frequently observed FokI, ApaI, and TaqI polymorphisms [51]. In our study, we also detected FokI, TaqI, and ApaI polymorphisms among the gene regions we examined.

In a meta-analysis and systematic review published in 2021, the results of ApaI, FokI, TaqI, BsmI, and BglI polymorphisms were evaluated, and it was reported that only the FokI polymorphism had a significant association with dental caries [52]. In another study, a possible relationship between the FokI polymorphism and dental caries was also suggested [53]. In a different study, no association was found between *VDR* gene polymorphisms (FokI and BglI) and serum vitamin D levels with dental caries [54]. In our study, the FokI polymorphism was not observed in only 2 out of 26 patients, and the ApaI polymorphism was detected in both alleles in 52% of the patients and in one allele in 40% of the patients. When the ratios between the caries-free and caries groups were compared, the results were similar. The statistical analyses did not identify a significant association between dental caries and these polymorphisms.

Various studies have investigated the association between BsmI and ApaI polymorphisms and dental caries [52]. Along with this, literature reviews have revealed a study demonstrating the association between the BsmI polymorphism and dental caries [55]. In our study, the ApaI polymorphism was not observed in three of the 26 patients, and the ApaI polymorphism was detected in a single allele in 61.6% of all the patients and in two alleles in 26.9%. When the ratios between caries-free and carious groups were examined, the results were similar. Statistical analyses did not identify a significant association between dental caries and these polymorphisms.

In another meta-analysis, a significant difference was found between the TaqI polymorphism and dental caries [56]. In a different study, a possible relationship between dental caries and the TaqI polymorphism was demonstrated, but no relationship was found with other polymorphisms [13]. Another study also reported a possible link between the TaqI polymorphism and dental caries [57]. However, further research suggested that while the TaqI polymorphism did not affect caries incidence, the *VDR* gene might still influence the caries formation process [58]. Additionally, a separate study found no significant relationship between the *VDR* gene TaqI polymorphism and dental caries [59].

## 5. Strengths

Selection of Participants: This study focused on children aged 3–6 years during the primary dentition period to ensure standardization and minimize variability related to tooth development.

Seasonal Data Collection: Data collection was restricted to winter months to control seasonal variations in vitamin D levels and ensure consistency across the participants.

Ethnic Homogeneity: This study was conducted in a specific region of Turkey, which provided the advantage of ethnic homogeneity. Future research should consider including diverse ethnic groups to explore genetic variations.

Inclusion Criteria: Children with chronic diseases or physical disabilities that could affect blood values or oral hygiene were excluded to avoid confounding factors.

## 6. Limitations

Cross-Sectional Study Limitations: As a cross-sectional study, causality between vitamin D levels and dental caries could not be established. The relationships observed may be less reliable than those found in cohort studies.

Post Hoc Power Analysis: The power analysis indicated a decrease to 63% for one-tailed and 50% for two-tailed tests. Future studies should consider larger sample sizes to detect smaller effects.

Genetic Analysis: Our study utilized Sanger sequencing to identify gene variations, which resulted in a limited sample size. Nonetheless, this approach confirmed that the *VDR* gene is well-conserved, as no mutations were detected in either the caries-free or carious children.

Potential Bias: The possibility of bias was acknowledged, as children with dental complaints prior to this study may have received more oral care advice, potentially affecting results.

## 7. Conclusions

This study found no direct, statistically significant relationship between vitamin D deficiency or insufficiency and dental caries in children aged 3–6 years. While higher vitamin D levels were observed in the caries-free group, the difference was not statistically significant. Genetic analysis revealed no mutations in the *VDR* gene, and no direct correlation between vitamin D levels and other biochemical data was established. Despite the absence of a clear connection between vitamin D and caries, the importance of preventive oral health education for parents was emphasized. Future research should further explore the potential role of vitamin D supplementation, include larger and more diverse participant groups, and focus on preventive strategies for dental health.

## Figures and Tables

**Table 1:** Reference ranges used for serum vitamin D concentration [11,14,18,19,20].

Baseline Reference Range 1		Ege University Faculty of Medicine Reference Range 2	
Status	Serum 25(OD)D level	Status	Serum 25(OH)D level
severe deficiency	0–9 ng/mL	significant deficiency	10 ng/mL and below
deficiency	10–19 ng/mL	mild–medium deficiency	10–19 ng/mL
suboptimal	20–29 ng/mL	optimal	20–50 ng/mL
optimal	30–49 ng/mL	increased risk level for hypercalciuria	51–80 ng/mL
high	≥50 ng/mL	possibility of toxicity	80 ng/mL and above

**Table 2:** Primers used for amplifying the coding exons and the exon–intron boundaries of the *VDR* gene.

Primers		Sequence (5′-3′)
3	forward	GCACCAAGGATGCCAGC
	reverse	CCTTCATGGAAACACCTTGC
4	forward	GTGATGACAGGGTGAGGAGC
	reverse	AAGGCCTTTCCCTGACTCC
5	forward	AAGGTTTCCTGGAGGAGCTG
	reverse	CCCTCTGTCCCTACTCCCTG
6	forward	ATCAGGGCCAAGGTAGGAAG
	reverse	GTGCGGTGGACTCCTCG
7	forward	CAGAGGGAAGCCTGGGGCT
	reverse	GTGGTGGATGAGTGATCTCCAACCC
8/9	forward	TGATTTGTGTGGCTTGAAGG
	reverse	TTTGTCCTTCATACTCCCCG
10	forward	GGTGGTGGGATTGAGCAG
	reverse	ACGTGGCCCTGGAGGAG

**Table 3:** Characteristics of children included in this study.

		Caries-free (n = 64)		Caries (n = 64)			
Variables	Classification	n	(%)	n	(%)	t	*p*
Age (years)	3	14	21.9	2	3.1	Chi-square trend = 28.949 *	0.000
	4	19	29.7	8	12.5		
	5	19	29.7	12	18.8		
	6	12	18.8	42	65.6		
Sex	female	32	50.0	32	50.0	Chi-square = 0.000 **	1.000
	male	32	50.0	32	50.0		
Frequency of tooth brushing (per day)	<2	44	68.7	45	71.3	Chi-square = 1.368	0.505
	2≥	20	31.3	19	29.7		
Who brushed the teeth	by child alone	32	50.0	35	54.7	Chi-square = 0.407	0.816
	with family	23	35.9	22	34.4		
	by family	9	14.1	7	10.9		
Frequency of routine dental visits (6 month)	yes	1	1.6	7	10.9	Chi-square = 4.879	0.087
	more than 6 months	10	15.6	8	12.5		
	no	53	82.8	49	76.6		
Fluoride treatment (in the last 6 months)	yes	1	1.6	1	1.6	Chi-square = 0.000	1.000
	no	63	98.4	63	98.4		
Use of fluoridated toothpaste	yes	22	34.4	45	70.3	Chi-square = 16.568	0.000
	no	42	65.6	19	29.7		
Sugar consumption between meals	never	8	12.5	1	1.6	Chi-square = 8.098	0.017
	rarely	29	45.3	24	37.5		
	every day	27	42.2	39	60.9		
Measurement serum vitamin D level	yes	15	23.4	13	20.3	Chi-square = 0.183	0.669
	no	49	76.6	51	79.7		
Taking a vitamin D supplement	yes	23	35.9	13	20.3	Chi-square = 3.865	0.049
	no	41	64.1	51	79.7		
Daily milk, cheese, egg, and fish consumption	Does not eat	8	12.5	5	7.8	Chi-square = 5.824	0.054
	rarely	4	6.3	13	20.3		
	eats at least one every day	52	81.3	46	71.9		

The data are presented as mean with 95% CI; * *p* values were determined from complex samples chi-square trend tests; ** *p* values were determined from complex samples chi-square tests.

**Table 4:** Serum 25(OH)D, HGB, Ca^+2^, P^−3^, and ALP status by caries-free and caries groups.

		Caries-free (n = 64)		Caries (n = 64)			
		n	(%)	n	(%)	t	*p*
25(OH)D Level (two classifications)	deficiency and suboptimal (3–29 ng/mL)	50	78.10	55	85.90	Chi-square = 1.325	0.250
	optimal (30–50 ng/mL)	14	21.90	9	14.10		
25(OH)D Level (three classifications)	severe deficiency (0–9 ng/mL)	7	5.50	9	7.00	Chi-square = 0.888	0.642
	deficiency (10–19 ng/mL)	29	22.70	32	25.00		
	optimal (20–50 ng/mL)	28	21.90	23	18.00		
HGB	low (<11.5)	15	23.40	14	22.20	Chi-square = 0.027	0.870
	normal (11.5–14.5)	49	76.60	49	77.80		
Ca^+2^	normal (8.6–10.2 mg/dL)	57	89.10	62	96.90	Chi-square = 2.988	0.84
	high (>10.2 mg/dL)	7	10.90	2	3.10		
P^−3^	normal (3.1–6)	64	100.00	63	98.40	Chi-square = 1.008	0.315
	high (>6)	-	-	1	1.60		
ALP	low (<142 IU/mL)	5	7.80	5	7.80	Chi-square = 1.009	0.604
	normal (142–335 IU/mL)	59	92.20	58	90.60		
	high (>335 IU/mL)	-	-	1	1.16		

**Table 5:** Correlation between vitamin D and other variables.

		Vitamin D
dmft	Spearman rho	−0.151
	p	0.088
Age	Spearman rho	−0.127
	p	0.153
HGB	Spearman rho	−0.075
	p	0.403
Ca^+2^	Spearman rho	0.115
	p	0.196
P^−3^	Spearman rho	−0.088
	p	0.322
ALP	Spearman rho	−0.119
	p	0.181

**Table 6:** Numerical comparison of polymorphisms seen in caries-free and carious groups. (According to the NM_000376.3 transcript.)

	Result of the Exon-3 (FokI Polymorphism)				Result of the Exon-10 (TaqI Polymorphism)				Result of the Exon–Intron Junctions-10 (ApaI Polymorphism)			
	Caries-Free *		Caries		Caries-Free		Caries		Caries-Free		Caries	
	n	%	n	%	n	%	n	%	n	%	n	%
Normal	1	4.0	1	4.0	3	11.5	3	11.5	1	3.8	2	7.7
Heterozygous polymorphism	3	12.0	7	28.0	10	38.5	6	23.1	10	38.5	6	23.1
Homozygous polymorphism	8	32.0	5	20.0	0	0.0	4	15.4	2	7.7	5	19.2
Total	12	48.0	13	52.0	13	50.0	13	50.0	13	50.0	13	50.0

* The examination in question 3 was performed with 12 caries-free children.

## Data Availability

The original contributions presented in the study are included in the article, further inquiries can be directed to the corresponding author.

## Abbreviations

*VDR*: vitamin D receptor; PCR: Polymerase Chain Reaction; Ca^+2^: calcium; P^−3^: phosphorus; ALP: alkaline phosphatase; HMG: hemoglobin; 25(OH)D: 25-hydroxyvitamin D, calcidiol; 1,25(OH)_2_D): 1,25-dihydroxy vitamin D, calcitriol; dmft: index, where the d component is the number of teeth with active decay, the m component is the number of missed teeth, and the f component is the number of teeth with filling; OR: odds ratio; CBC: complete blood count; EDTA: Ethylenediaminetetraacetic Acid; LC/MS: liquid chromatography–tandem mass spectrometry; HGMD: The Human Gene Mutation Database.
